# End-of-life dreams and visions in a patient with delirium: A Brazilian case report and narrative review

**DOI:** 10.1017/S1478951525101247

**Published:** 2025-12-26

**Authors:** Taís Oliveira Silva, Bruno Angeli-Faez, Lorena Cândida Ferreira Paixão, Everton de Oliveira Maraldi, Alexander Moreira-Almeida

**Affiliations:** 1 Institute for Human Sciences, Psychology Department, Federal University of Juiz de Fora, Minas Gerais, Brazil; 2 Research Center in Spirituality and Health (NUPES/UFJF), School of Medicine, Federal University of Juiz de Fora, Minas Gerais, Brazil; 3 University Network for Research in Spirituality (REUPE), Institute of Health Sciences, Federal University of Bahia, Salvador, Brazil; 4 Research Program on Non-Ordinary Experiences and Altered States of Consciousness (PROEX), Institute of Psychiatry, Clinical Hospital - University of São Paulo, São Paulo, Brazil; 5 Postgraduate Program in Medical Sciences at the D’Or Institute for Research and Teaching (IDOR), Rio Janeiro, Brazil

**Keywords:** End-of-life dreams and visions, end-of-life experiences, delirium, CAM, palliative care

## Abstract

**Objectives:**

End-of-life dreams and visions (ELDVs) and delirium frequently occur near death but differ in core features. Clinical differentiation becomes challenging when they co-occur. This case report illustrates the interplay between ELDVs and delirium, examines the limits of current diagnostic criteria in mixed cases, and outlines a nuanced approach to distinction.

**Methods:**

We report the case of an elderly Brazilian woman with metastatic cancer who exhibited both ELDVs and delirium. Mental status was serially assessed using the Confusion Assessment Method (CAM). ELDV accounts were prospectively triangulated across patient, family, and clinician reports to enhance reliability and contextual understanding.

**Results:**

The patient’s experiences showed ELDV hallmarks – vivid, realistic encounters with deceased relatives, biographical relevance, and preparatory themes – yet many were affectively distressing and occurred alongside fluctuating attention and consciousness consistent with delirium. CAM effectively identified delirium but could not, on its own, distinguish ELDVs within delirious states. When co-occurring, ELDVs often emerged during “windows of lucidity” marked by preserved autobiographical context, intact recall with subsequent coherent narration, and insight, despite intense emotional valence. Distress alone was not discriminatory, probably being shaped by psychosocial and cultural factors. These observations indicate the need to supplement CAM with qualitative and phenomenological criteria, including content, vividness, biographical meaning, insight, cultural fit, and acuity/recall.

**Significance:**

To our knowledge, this is the first case to map evolving end-of-life mental status using serial CAM while prospectively documenting ELDVs via triangulated reports. The findings highlight the complexity of differentiating co-occurring ELDVs and delirium and challenge the sufficiency of CAM alone. An integrated approach – combining CAM screening with structured ELDV assessment – may prevent misclassification and support holistic, dignified palliative care. As a single case in an underexplored domain, these insights require confirmation in larger, prospective studies to assess generalizability.

## Introduction

End-of-life dreams and visions (ELDVs) and delirium are frequently observed phenomena in patients with advanced, life-limiting illnesses (Ijaopo et al. 2023). While both are common in the context of advanced disease, they are considered to represent distinct clinical entities (Depner et al. 2020). ELDVs refer to meaningful mental and sensory experiences that occur either during sleep (dreams) or while awake (visions) (Depner et al. 2020), often featuring encounters with deceased loved ones shortly before death (Kerr et al. 2014; Grant et al. 2023). In contrast, delirium is regarded as a multifactorial organic neuropsychiatric condition marked by a sudden onset and fluctuating impairments in attention, consciousness, cognitive abilities (memory, orientation, language, and perception), and disturbance of the sleep-wake cycle (Maldonado 2018; Guo et al. 2021). The challenge for clinicians arises when these distinct phenomena co-occur, demanding careful differentiation to ensure appropriate clinical management.

Reported prevalence of ELDVs varies widely – from 50% to 88.1% – depending on methodological differences, diagnostic criteria, and clinical settings, such as hospice, nursing homes, acute care and home-based palliative care (Hession et al. 2022, 2024; Silva et al. 2023). These variations may reflect differences in cultural context and willingness to report such experiences (Rabitti et al. 2024). Despite increasing empirical interest, ELDVs remain underrecognized in clinical practice and are sometimes misinterpreted as symptoms of delirium (Depner et al. 2020; Grant et al. 2020; Silva et al. 2023).

Reports of ELDVs span various historical periods (Ethier 2005; Betty 2006; Santos and Fenwick 2011) and cultural contexts (Hession et al. 2022; Silva et al. 2023), highlighting their widespread nature. Research findings have challenged the assumption that ELDVs are merely byproducts of medication side effects or delirium (Kerr et al. 2014; Depner et al. 2020; Grant et al. 2020), emphasizing their unique characteristics and potential clinical significance.

Delirium is also a widespread occurrence at the end of life, affecting between 28% and 42% of patients upon admission, with prevalence rising to 88% near death (Hosie et al. 2013; Watt et al. 2019). Unlike the cognitive clarity often associated with ELDVs, delirium fundamentally involves fluctuating cognitive impairment (Kerr et al. 2014; Depner et al. 2020). Patients experiencing ELDVs typically retain the ability to provide coherent and detailed narratives that convey a sense of realism, often describing these experiences as vivid and lifelike (Mazzarino-Willett 2010; Nosek et al. 2015; Depner et al. 2020). However, ELDVs can, at times, occur concurrently with episodes of delirium. Notwithstanding, some authors suggest that even in these cases, ELDVs generally occur in windows of lucidity, involving a clear consciousness, heightened awareness, and acute environmental perception (Kerr et al. 2014). These experiences are vivid, memorable, subjectively meaningful, and often provide personal comfort, contrasting with the distressing nature of delirium alone (Kerr et al. 2014; Kerr 2022).

Unlike delirium, which often causes agitation and fear (Maldonado 2018; Guo et al. 2021), ELDVs are commonly associated with feelings of comfort and a sense of acceptance toward death (Kerr et al. 2014; Depner et al. 2020). For instance, a longitudinal study of 59 American patients revealed that 60.3% found their ELDVs comforting or extremely comforting, with those involving deceased loved ones offering greater comfort than those of living individuals (Kerr et al. 2014). Additionally, some ELDV studies exclude patients with a history of delirium, employing strict screening to distinguish these experiences (Kerr et al. 2014; Depner et al. 2020; Levy et al. 2020a; Nyblom et al. 2021).

Nevertheless, a relatively smaller subset of American patients reports distressing ELDVs (18.8%) (Kerr et al. 2014); often linked to unresolved traumas or unfinished business (Nosek et al. 2015; Shinar and Marks 2015). A similar prevalence was found in a Japanese study about deathbed visions (DBV), where 19% of family members agreed or strongly agreed that their loved ones appeared fearful or uneasy in response to their DBVs (Morita et al. 2016). Although challenging, these distressing experiences can be transformative, helping patients address lingering issues by alleviating guilt and facilitating forgiveness. (Nosek et al. 2015; Shinar and Marks 2015; Kerr 2022; Claxton-Oldfield 2023). Overall, the findings suggest that ELDVS foster profound inner insights, contribute to posttraumatic growth (PTG), and enhance the psychological and spiritual well-being of the dying (Levy et al. 2020a; Rabitti et al. 2024). However, the mechanisms underlying distressing versus comforting ELDVs remain underexplored (Claxton-Oldfield 2023).

Therefore, key differentiation criteria include (Depner et al. 2020; Grant et al. 2023): (1) phenomenological clarity: ELDVs are typically clear and coherent, even if interspersed with delirium. (2) Content specificity: ELDVs often feature deceased loved ones, travel, or transition themes, distinct from generalized delirium hallucinations. (3) Biographical relevance: ELDVs are often deeply personal and meaningful to the patient. (4) Emotional valence: while ELDVs can be distressing, they often provide comfort or insight, unlike the pervasive fear of delirium. (5) Insight and recall: patients often retain insight into ELDVs and can recount them coherently during periods of lucidity.

Despite the recognized clinical importance of both delirium and ELDVs in end-of-life care, research exploring their potential co-occurrence and interplay remains scarce. This knowledge gap leaves clinicians ill-equipped to care for affected patients effectively. Misidentifying ELDVs as merely delirium and treating them solely with pharmacological interventions could inadvertently deprive dying patients of meaningful and comforting experiences vital for enhancing the quality of their end-of-life process (Kerr et al. 2014; Silva et al. 2023). Conversely, if patients experience both delirium and ELDVs, failing to medicate delirium might hinder their ability to access the comfort and potential for PTG that ELDVs seem to provide (Levy et al. 2020a). Therefore, further research is urgently needed to elucidate the relationship between delirium and ELDVs and to inform best practices for their nuanced clinical assessment and management.

### Aims of the study

This article aims to present and critically analyze a case study that illustrates the complex interplay and potential co-occurrence of ELDVs and delirium in a terminally ill patient. Through this analysis, the study seeks to stimulate an in-depth discussion regarding the precise classification and comprehensive understanding of these phenomena and the clinical challenges involved in their co-occurrence. This specific case warrants particular attention due to the notable diagnostic overlap observed between ELDVs and delirium, a conflation corroborated by prospective reports triangulated from the patient, their family, and the clinical team, in conjunction with systematic Confusion Assessment Method (CAM) assessments. Consequently, the inherent diagnostic complexity exemplified by this case serves as a significant representation of the possible ambiguity encountered by healthcare professionals in palliative care settings.

Beyond illustrating these phenomena, this report seeks to draw attention to and encourage systematic investigation into the potential co-occurrence of ELDVs and delirium within terminally ill patient populations. By highlighting these complexities, this study underscores the critical need for a nuanced approach to differential diagnosis and clinical management, thereby preventing misidentification that could lead to inappropriate interventions and ultimately enhancing holistic patient care.

The patient and her family were participants of a larger study of end-of-life experiences (ELEs) among terminally ill patients in Brazilian palliative care units. Interviews were conducted by the first author at the patient’s daughter’s home. The researcher accompanied the home-based palliative care (PC) team during their visits to provide care. To minimize bias and ensure confidentiality, interviews were conducted independently and reports collected within 48 hours of each occurrence. Triangulation of patient, family caregiver, and clinician reports enhanced the study’s validity and provided a more comprehensive understanding of the observed phenomena.

The patient’s mental state at data collection moment was systematically assessed and monitored using the CAM, a well-established tool for diagnosing delirium (sensitivity of 94%–100% and specificity of 90%–95%) (Inouye et al. 1990; Fabbri et al. 2001), already validated in PC settings (Ryan et al. 2009). According to the CAM algorithm, a delirium diagnosis requires the presence of both the first criterion (acute onset or fluctuating course) and the second criterion (inattention), along with either the third (disorganized thinking) or fourth criterion (altered level of consciousness) (Inouye et al. 1990).

In the subsequent case description, pseudonyms are used to protect the identities of the patient and her family, and all potentially identifying details have been omitted to ensure anonymity.

## Case description

Mrs. Dayane, a 61-year-old retired widow with 2 daughters, was diagnosed with lung cancer and bone/central nervous system metastases. Referred to a home-based PC to manage severe symptoms, including headache, nausea, immobility, and dyspnea, she relocated to her eldest daughter’s home in a low-income neighborhood in southern Brazil. Her sister also provided care. Prior to admission, she experienced seizures, which were controlled with anticonvulsants and corticosteroids. Despite being very thin and bedridden due to disabling symptoms, multidisciplinary care led to improvement in her pain, nausea, loss of appetite, and sleep.

Mrs. Dayane had a history of depression and expressed suicidal ideation during follow-up. Her eldest daughter reported the patient’s profound fear of dying, as her mother had 44 years prior, who also suffered from the same diagnosis. This fear prompted the patient to express a desire to overuse medications, leading her daughter to remove them as a safety precaution. This history of emotional vulnerability provides important context for understanding her subsequent experiences.

The patient was followed by the home-based PC team, with variable visit frequency, for 46 days in the last quarter of 2021, before her daughters opted for hospital transfer. From the 12th day to the 36th day of follow-up, her mental state was monitored with CAM on 13 visits. In the initial interview, her eldest daughter reported that 3 months prior to homecare admission, the patient had dreamed about her own deceased mother, an experience her daughter interpreted as comforting and an unconscious effort by the patient to come to terms with her situation.

During the first home visit, Mrs. Dayane identified as Catholic and Evangelical Lutheran, stating she believed in God and that human beings are composed of a body and a soul/spirit, but held no opinion about life after death or reincarnation. Her main family caregiver shared similar beliefs, while her sister was an Evangelical Lutheran.

On the 25th day of follow-up, the patient was not experiencing delirium (CAM−) and evidenced controlled pain and nausea. At this moment, she reported her first dream experience. She reported a dream about a living niece who had given birth. *“Then, when I picked up [the baby] … I woke up with her in my arms.”* She felt profound happiness, interpreting it as a “sign *of more life for me… And hope.”* This episode indicates a typical ELDV reported in a state of cognitive clarity.

Her eldest daughter reported that Mrs. Dayane’s condition worsened after receiving news of her irreversible disease from the oncologist. Days later (days 28 and 29 of follow-up), she experienced both ELDVs and delirium, prompting the home-based PC team to administer low-dose antipsychotics therapy for delirium management. On day 28, delirium was confirmed by a positive CAM screen: she was absent throughout the interview, lethargic, and sleeping most of the time, thereby fulfilling CAM criteria 1, 2, and 4. On the preceding night, the eldest daughter reported that the patient had been agitated and appeared to be conversing with deceased loved ones – her late mother, ex-sister-in-law, and even her childhood teachers, so she took the patient to the emergency room. During this episode, which displayed features of an ELDV occurring within a delirious state, the patient referred to past experiences, saying, *“Mom, let me open the door, I have to go,”* and she described intense fear of dying. Her daughter interpreted the calls for her mother as a connection to the shared disease that had caused her mother’s death. This instance suggests the co-occurrence of ELDVs and delirium, where the ELDV content and characteristics – including vivid, intense conversations with deceased relatives – remained phenomenologically distinct despite the altered consciousness associated with delirium.

On the 29^th^, during the interview, the patient was CAM positive. She was markedly lethargic, though present at some time in a marked form, and also exhibited psychomotor retardation, thereby meeting criteria 1, 2, 4, and 9 of the CAM algorithm. At the outset, the home-based PC psychologist observed a significantly reduced level of consciousness, characterized by pronounced drowsiness. Nevertheless, upon recounting her experience, Mrs. Dayane became notably lucid, as stated by the psychologist, “*She said that she had died that night, and that she had come back from that death. That she had, let’s say, been resurrected, right?”* When asked for an explanation about the patient’s experience, the psychologist speculated that *“maybe she really did… go through that process. That she really did, uh …, how she really felt, she said that she even commented that it wasn’t something that pleasant, right.”*

The psychologist further reported that the patient required the presence of relatives, including a sister from another city, and a pastor, commenting: *“So, in a way it seems that she is preparing herself [for the dying process], making all the necessary arrangements, you know?.. with her family… for her departure.”* The patient also expressed significant fear and a strong aversion to recounting or reliving the experience of apparent death, complaining of frequent nightmares, accompanied by loud screams. When asked if assistance could reduce her fear, she declined, affirming, “*I impose a lot of fear on myself.”*

Later that same day, the patient continued to exhibit transient confusion, momentarily failing to recognize her own daughter. During medication administration, she cried out, *“Ouch .. ouch .. I’m going to fall.”* Her eldest daughter reported that the patient continued speaking to her deceased mother and husband and referred to *“a sister of hers who had passed away, who had gone to see her, and that she wanted her to come here, to the house. And that she wanted to know how she was going to get up.. She used the term ‘get up’.”* The daughter confirmed the patient’s request for a pastor, *“because.. she seems to want to say goodbye and is worried about the afterlife.”* She also noted that the patient voiced fear of dying and stated that, even if she were to go to a good place after death, she was not yet ready to die. Although these phenomena occurred during fluctuating periods of delirium, they nonetheless contained themes and elements characteristic of ELDVs, including interactions with deceased loved ones, vividness, intensity, and motifs of transition.

On the same day, the patient’s sister reported that Mrs. Dayane was afraid of dying and did not want to die alone, inviting her sister to accompany her. She added that the patient asked about another deceased sister. However, when Mrs. Veronica replied that she had become “*a little star,”* the patient categorically denied this statement. The sister corroborated the requests for a pastor and the patient’s reported “death” experience. Despite evident distress and anguish, Mrs. Veronica noted a sense of acceptance in her sister, suggesting both preparation for death and reassurance. She also observed a significant reduction in the patient’s reported pain, and wondered whether these experiences might represent medication-induced hallucinations. These familial observations provide invaluable qualitative data that complement the objective CAM assessments and illuminate the patient’s subjective experience.

On the 32^nd^, her eldest daughter reported that the previous night, the patient *“hallucinated, was quite delirious, became quite agitated… I think she slept for a maximum of five* *minutes and woke up talking.”* The patient engaged in spontaneous, seemingly conversational dialogues, as if speaking with her siblings, raising her hands toward the ceiling. She uttered fragmented phrases, such as “*I don’t know what, Duda.*..,” likely referring to a deceased sister. While some speech was incomprehensible, the daughter observed a connection to childhood memories. By the subsequent morning, at the time of the interview, the patient was awake and fully lucid, with a negative CAM score. This rapid fluctuation suggests either the dynamic nature of her delirious state or that the daughter’s initial interpretation of the patient’s ELDVs was perceived as an episode of delirium.

The patient told her youngest daughter (Mrs. Laura) that she was talking to a child with curly hair. The latter thought the patient might be remembering her eldest sister as a child, given her curly hair, but the patient affirmed that she was referring to “another girl.” At one point, while looking at the wall, the patient said, *“Oh, the animals are coming to get me.”* Despite these perceptual alterations, her eldest daughter acknowledged that the patient was calmer and speaking more clearly than in previous days and was again recognizing her family members. This dynamic presentation highlights how features consistent with ELDV (visions of a child, coherent conversations about it, and heightened tranquility) can intersperse with more typical delirium-associated hallucinations (animals coming to get her).

On the 33rd day, CAM screening was not feasible because the patient was asleep. Her sister corroborated the unusual experience reported the previous day. Mrs. Dayane appeared to be interacting with a child, describing “sawing” and “carrying” a curly-haired child who, she claimed, was swinging and laughing at her; she behaved as if caressing the girl. On the 36th day (CAM+), she was present at some time during the interview, but in mild form, lethargic and with psychomotor retardation, meeting CAM algorithm criteria 1, 2, 4 and 9), the patient’s sister reported that during the night the patient had called to and spoken with her deceased sisters, saying “come…come,” again extending her hands up, and later saying “no.no…no….” The sister added, “*Then later, we asked her ‘what happened?’ She answered, ‘There, there, she is.’ Then she smiled, and soon, she passed out*.” The sister also reported that the patient’s anxious behavior occurred more frequently at night, when she repeatedly called for her. During the day, the patient was calmer, but did not want to be alone. Intermittent CAM assessments throughout follow-up revealed the waxing and waning course of delirium, which at times coincided with ELDV reports; however, CAM alone was insufficient to fully capture the subjective nuances of these complex experiences, including the specific content of dreams and visions and their impact on the patient‘s overall mental state.

According to her eldest daughter, from day 36 to 46, the patient remained confused and aggressive, especially at night, experiencing fear of being alone and insomnia. The family therefore decided to admit her to a local hospital, where the patient died 4 days later, reportedly in her sleep, according to her eldest daughter, who was uncertain whether the patient had been sedated in her final days or experienced additional ELDVs while hospitalized.

A record of all ELDVs reports in this case is presented in Supplementary Material I alongside the CAM monitoring data.

## Discussion

To the best of our knowledge, this is the first case report to comprehensively describe the evolving mental state changes during the end-of-life phase, using a widely recognized delirium assessment tool, and incorporating prospective ELDV reports from the patient, family members, and clinicians (see Supplementary Material I).

The patient experienced both ELDVs and delirium prior to death. In the absence of a standardized instrument for ELDVs, unlike delirium, which is assessed using CAM, our discussion centers on (1) the salient features of the observed ELDVs and (2) the interplay between the patient’s ELDVs and her emotional responses during episodes of delirium (indicated by CAM+).

### Characteristics of observed End-of-life dreams and visions (ELDVs)

The patient’s dreams and visions displayed hallmark features consistent with established descriptions of ELDVs. Qualitative research with ELDV subject-matter experts characterizes these phenomena as personal, subjective experiences intrinsic to the dying process; they are typically extremely vivid, may occur during sleep or wakefulness, and often provide meaning and/or comfort that aids coping with the transition from life to death (Grant et al. 2023). Core hallmarks include realism, meaning/comfort, and characteristic content such as visits from deceased relatives and friends, travel motifs, and pets (Grant et al. 2023).

Below, we correlate the patient’s experiences with these established hallmarks:

1. Typical contents: A predominant theme was interaction with deceased family members and friends. Mrs. Dayane frequently mentioned seeing many deceased relatives and acquaintances (Kerr et al. 2014; Nosek et al. 2015; Dam 2016; Pan et al. 2021) – “*She kept talking to her grandmother, and today she spoke about a sister of hers who had passed away and had come to see her, and that she wanted her to come here, to her house.”* Her experiences also featured typical ELDV themes, such as references to travel, which involved preparing to go: *“Mom, let me open the door, I have to go;” “she wanted to know how she was going to get up”* (Nosek et al. 2015; Depner et al. 2020; Nyblom et al. 2021). Additionally, her ELDVs notably lacked religious content as described in previous studies in Western cultures (Kerr et al. 2014; Depner et al. 2020; Nyblom et al. 2021). This is curious given the significant role played by religiosity/spirituality in Brazilian culture (Maraldi et al. 2021). This may suggest that the content of ELDVs is relatively stable across cultures, despite the patient‘s beliefs and expectations – we will return to this later in the section on cultural differences. Still, it is important to note that belief in life after death (Curcio and Moreira-Almeida 2019) and anomalous perceptions involving deceased people (i.e., bereavement hallucinations) are highly prevalent in the general Brazilian population (Monteiro de Barros et al. 2025), with potential implications for Mrs. Dayane ELDVs. For example, the themes of transition, conversations with deceased loved ones, and the experience of dying and then returning to life are consistent with Spiritualist accounts of the afterlife. Although Mrs. Dayane does not recognize herself as a spiritualist, belief in spirits and the afterlife is widespread in Brazilian culture, going beyond their adherence by spiritualist believers (van der Hoek 2024).
2. Realism: ELDVs are frequently described by patients as *“more real than real”* (Kerr 2022, p. 7). Although this patient didn’t explicitly comment on their reality or contrast them with other mental states, her experiences were perceptually distinctive and immersive, blurring the boundary between observer and unfolding events. Rather than mere recollections, they were vivid, detailed, and intense firsthand encounters that felt highly realistic – *“when I picked up [the baby] … I woke up with her in my arms;” “I died, right? Say it’s a lie that I died.”* The sense of reality can thus be inferred from her emotional responses and attitudes toward these experiences.
3. Meaning and/or comfort: Although distressing, the patient’s experiences appeared to facilitate new insights and understanding for the patient, demonstrating that the dying person is not merely passive or constrained by physical decline, but actively engaged as a negotiator or co-participants in the events encountered (Kerr 2022; Silva et al. 2023). Illustrative statements include: *“I impose a lot of fear on myself”; “I don’t know, I don’t know if you’re going with me.” The* patient’s sister said *“I’m going [with you], I’ll go to the door and hand it over to you.”* The patient said, *“No, no, I don’t want to go with you, I want you to go with me.”* Her sister asked, *“How are we going?”* The patient answered, *“Well, it’s up to God to know how you’re going.. But I’ll only go if you go, don’t leave my side”* (Supplementary Material I). Furthermore, her experiences appeared to serve an adaptive function, facilitating psychological preparation for death and addressing unfinished matters (Kerr et al. 2014; Nosek et al. 2015; Levy et al. 2020b): *“she called all her family … All her brothers, she was afraid.. That her sister who lives a little further away wouldn’t arrive, because she said several times that her sister was taking a long time to arrive. She asked a pastor to arrive in the afternoon… To be able to talk to her… So, in a way it seems like she is preparing herself, and making all the necessary arrangements, you know?”*

### Interplay with delirium and differential diagnosis

Distinguishing ELDVs from delirium presents a significant clinical challenge when they experience both phenomena concurrently. While the literature outlines key elements for differentiation (see Table 1 in Depner et al. 2020, p. 104), our case highlights that while ELDVs are typically coherent and meaningful, they can manifest concurrently with the fluctuating states of consciousness and disorientation characteristic of delirium. The critical distinction lies in the qualitative difference of the perceptual experience itself: ELDVs, even within a delirious state, may retain a subjective sense of clarity and purpose, preparing to death, contrasting sharply with the fragmented nature of purely delirious hallucinations.

Employing Depner et al.’s (2020) distinct criteria across various “Spheres of Being” to differentiate these phenomena, we observed instances where Mrs. Dayane’s experiences diverged from typical ELDV characteristics, particularly within cognitive and emotional spheres. These divergences manifested as distressing episodes occurring alongside fluctuating states of consciousness and attention, coupled with sleep-wake cycle disturbances – hallmarks of delirium (Maldonado 2018). Specifically, the patient presented mixed delirium – mostly fluctuant states of hyperactive delirium during the night and of hypoactive delirium during the day. Mixed delirium subtype seems to have greater symptom burden than both hypoactive or hyperactive subtypes (Glynn et al. 2021).

**Cognitive state:** The CAM effectively diagnosed delirium in Mrs.Dayane through criteria indicating “Disorganized/Confused” cognitive states, including “fluctuating consciousness and attention” and “altered states of consciousness” (Inouye et al. 1990). During delirious episodes, she was often “absent throughout the interview” and “markedly lethargic.” Conversely, during her ELDVs reports with CAM negative, she frequently exhibited preserved acuity and recall. For instance, after a dream about a baby, she provided a clear narrative, expressing “profound happiness” and interpreting it as a “sign of more life for me… And hope.” Notably, even during periods of CAM positivity, when recounting a “resurrection” experience, she became strikingly lucid, stating, “She said that she had died that night, and that she had come back from that death.” This remarkable clarity within narratives, even amid overall confusion, strongly aligns with the “Acuity/Recall” feature of ELDVs.

**Emotional state:** This sphere presented a critical point of divergence. While ELDVs are typically associated with feelings of “Comforted/Calm,” a significant portion of Mrs. Dayane’s experiences were “Extremely Distressing.” Her “intense fear of dying,” “frequent nightmares, accompanied by loud screams,” and expressions of fear strongly mirrored the distressed emotional state often linked with delirium. However, her initial ELDV with CAM negative involving the baby brought “profound happiness.” This complex emotional landscape suggests that while delirium may amplifiy distress, ELDVs provide comfort. Nevertheless, many experiences, particularly when co-occurring with delirium, were distressing. This observation underscores the challenge of using emotional valence alone as a definitive differentiator between both experiences (Machado and Moreira-Almeida 2021; Silva et al. 2023).

Delirium can be triggered by various pathological factors, whose effects vary according to individual physiological characteristics (Maldonado 2018). Mrs. Dayane had several delirium risk factors, such as advanced age, lung carcinoma with bone and central nervous system metastases, reduced physical mobility, sleep changes, opioid usage, as well as proximity to the end of life. The laboratory tests carried out in the beginning of her mental state’s changes, showed only a unnoteworthy slight increase in Lactate Dehydrogenase levels, meaning organ or tissue damage (Supplementary Material II).

To further differentiate ELDVs from delirium-associated hallucinations, we draw a comparison with a Japanese retrospective study by Tachibana et al. (2021). This study involving 602 patients (mean age 75.9 years) with diverse clinical conditions who developed delirium, reported that 25.9% (n = 156) experienced hallucinations during hospitalization. The majority were *“complex and concrete”* visual hallucinations (92.3% [*n* = 144]), such as:

“People (family and friends, *n* = 35; strangers, *n* = 61; Buddha, *n* = 2; the devil, *n* = 2; ghosts, *n* = 4; cadavers, *n* = 2); insects, *n* = 22; animals, *n* = 20; inorganic objects, *n* = 15; and visions, *n* = 26 (fire, *n* = 15; falling ceilings, *n* = 5; flowing water, *n* = 3; quarrels and wars, *n* = 3). Of these, the visual hallucinations especially strangers or extraordinary visions such as fire caused substantial anxiety and confusion. One subject saw a stranger, saying “he was creepy.” There was one subject who experienced simple, elemental visual hallucination, saying “I saw a big color” (Tachibana et al. 2021, p. 2).

The content and themes of these hallucinations were distinctly different from those of ELDVs. Notably, absent were reports of visions involving deceased relatives or friends, or transitions between realities. Only a minority of accounts contained transcendent elements (e.g., religious figures, ghosts, cadavers), with most visions centering on catastrophic events, such as fires, floods, and wars. This stark contrast between the general delirium-associated hallucinations and Mrs. Dayane’s experiences supports the argument that ELDVs possess a distinct phenomenology and are not merely manifestations of delirium. While Mrs. Dayane did experience some nonspecific hallucinations (e.g., “animals coming to get me”), the dominant and recurrent themes of deceased relatives and transitional journeys within her experiences align with typical ELDVs rather than the varied and often frightening content of typical delirium-related hallucinations. Although the generalizability of these findings to a specific end-of-life patient population may be limited, the advanced age and multiple comorbidities of most participants in the Japanese study underscore the importance of discerning the phenomenological differences between delirium-induced visions and ELDVs, particularly given the ongoing knowledge gaps regarding the prevalence, risk factors, and phenomenology of hallucinations in delirium (Tachibana et al. 2021).

The Tachibana et al. (2021) study also identified alcohol consumption, benzodiazepine withdrawal, and the use of angiotensin receptor blockers and dopamine receptor agonists as significantly associated with delirium-related hallucinations. Conversely, intrinsic factors (e.g., sensory impairments, psychiatric disorders, dementia) and environmental factors (e.g., ICU stays) showed no significant associations. In the present case, Mrs. Dayane had no history of alcohol use or abuse, nor was she receiving any of the medications identified as risk factors for hallucinations in the aforementioned study. She had also been receiving appropriate medical treatment for her mental state changes since their onset (days 28–29 of follow-up) (Supplementary Material III). While robust evidence is limited regarding pharmacological therapies for delirium in terminally ill adults, short-term, low-dose antipsychotics are generally recommended for managing perceptual disturbances, severe agitation, or safety concerns (Finucane et al. 2020; Bramati and Bruera 2021), with haloperidol typically being the first-line agent for delirium in advanced disease (Breitbart and Alici 2008). It is also important to consider that sedative medications may influence a patient’s consciousness, and their impact on spiritual experiences remains uncertain (Dalle Ave and Sulmasy 2025).

It is crucial to emphasize that the comparison between our case and the Japanese study may be shaped by underlying cultural differences. To date, relatively few cross-cultural investigations have systematically examined the phenomenology of ELDVs, highlighting the critical need to acknowledge how social, cultural, and contextual factors might influence these experiences. In the Brazilian context, for example, religiosity and spirituality play a central role in people’s worldviews and coping strategies, potentially shaping not only the frequency and content of ELDVs but also the meanings attributed to them (Maraldi et al. 2021). Additional factors such as socioeconomic conditions, disparities in healthcare access, and the broader social environment may further impact both the phenomenology and the mental health implications of such experiences in ways that remain insufficiently appreciated. Only through in-depth, systematic, and comparative research across different cultural settings will it be possible to clarify which aspects of ELDVs reflect universal features of human cognition and consciousness, and which aspects represent culturally specific expressions. Research on other anomalous and spiritual experiences, such as near-death experiences, has already demonstrated that cultural frameworks, beliefs, and expectations can significantly mold the phenomenology of these events, even though a set of cross-culturally recurrent features can also be identified (Shushan 2018; Maraldi and Krippner 2019).

### Psychosocial/cultural factors and emotional impact

Expanding on the discussion regarding psychosocial and cultural factors, it is crucial to note that following the diagnosis of an incurable disease and the realization of impending death, the patient’s emotional and mental state underwent a profound transformation. Her inner experiences, initially perhaps more neutral or even positive, gradually became increasingly distressing. These emotions appear to be linked not solely to her ELDVs, but also to her physical decline and the dying process. This underscores the significant role of psychosocial factors in shaping how individuals confront their mortality (Renz et al. 2012, 2018; Renz 2015).

Although some of her experiences, involving deceased loved ones and transitions between different realities (as illustrated by this statement: *“Tell me the truth… ‘that I did die, you’re crying…”*), were characterized by intense fear and anguish, these anxiety-inducing and fear-provoking episodes appeared to deepen her awareness of her imminent death. This, in turn, fostered insight and a sense of the need for resolution – typical features of distressing ELDVs (Kerr and Mardorossian 2020).

Scarce literature on distressing ELDVs often links them to past traumas, unresolved issues, or challenging relationships (Nosek et al. 2015; Shinar and Marks 2015; Kerr and Mardorossian 2020). According to her family caregivers, the patient’s mother’s suffering at death was a traumatic event, leading them to attribute the patient’s own distress to a profound fear of dying similarly. Compounding this, the patient had a history of depression and was described by her sister as prone to anger and resentment. Consistent with the notion that *“we die as we live”* (Kerr 2022, p. 26), it is reasonable to surmise that the patient experienced significant fear, with many of her ELDVs being interpreted/perceived as distressing. Her preexisting psychosocial vulnerabilities likely amplified the emotional intensity and negative valence of her ELDVs, thus contributing to her pervasive fear of death rather than the comfort often associated with these experiences.

Furthermore, the absence of strong spiritual beliefs held by the patient and her family, such as beliefs in life after death or reincarnation, which might provide a framework for understanding ELDVs involving deceased loved ones and transitions between different realities, combined with the lack of affiliation with a social group that supports and facilitates the incorporation of such experiences in a more beneficial way, may partially account for the fear and anguish associated with these experiences (Machado and Moreira-Almeida 2021; Mosqueiro et al. 2023; Silva et al. 2023; Maraldi et al. 2024).

Empirical evidence suggests that belief in life after death is linked to improved mental health outcomes, including lower levels of anxiety, depression, obsession-compulsion, paranoia, and social anxiety, as well as reduced symptom severity in conditions like phobia and somatization (Flannelly et al. 2006; Ellison et al. 2009; Bradshaw and Ellison 2010). It seems to foster tranquility, buffers the adverse effects of poor health and financial decline (Ellison et al. 2009), to moderate the impact of financial hardship (Bradshaw and Ellison 2010), and is associated with a belief in an equitable world (Flannelly et al. 2012). In terminally ill patients, this belief was associated with reduced end-of-life despair, hopelessness, suicidal ideation, and desire for hastened death (McClain-Jacobson et al. 2004). This contrasts with Mrs. Dayane’s experience, suggesting that the absence of a strong spiritual framework may have negatively influenced the nature and interpretation of her ELDVs, thereby depriving her of a crucial coping mechanism.

Therefore, cultural frameworks may shape not only the content of ELDV but also how patients interpret, integrate, and disclose these experiences, and how clinicians make sense of them. These influences are not merely background sociological variables; they have direct clinical and therapeutic salience, guiding whether an experience is stabilizing or destabilizing, and informing the most ethically and clinically appropriate response (Maraldi and Krippner 2019). Attending to these dimensions is essential for sensitive, culturally competent end of life care.

### Implications for assessment and care

This case highlights that while the CAM effectively diagnosed delirium, it proved insufficient in capturing the full phenomenological and emotional spectrum of the patient’s experiences, failing to inherently differentiate co-occurring ELDVs. This necessitated a nuanced clinical approach that integrated serial CAM assessments with careful attention to recognizing the core hallmark properties of ELDVs.

Consequently, our case experiences underscore that CAM remains the primary screening tool for delirium and should be used initially to establish its presence. However, as previously discussed, CAM positivity alone does not resolve whether concurrently reported dream/vision experiences are delirium-driven hallucinosis or ELDVs with distinctive end-of-life meaning.

For a nuanced clinical approach to managing the co-occurrence of these experiences, we suggest four sequential steps for clinicians:

- **Step 1:** Screen with CAM to identify delirium. This establishes the presence of delirium as the primary anchor.
- **Step 2:** If CAM positive, systematically assess against ELDV criteria. This involves evaluating the patient’s experiences based on phenomenology, biographical meaning, insight, cultural fit, and response to comfort measures.
- **Step 3:** If both are present, document as a dual process. This means acknowledging delirium with concurrent ELDV rather than attributing all phenomena solely to delirium.
- **Step 4:** Treat reversible delirium precipitants while simultaneously preserving, validating, and, when appropriate, gently facilitating ELDVs. This approach, following evidence-based guidelines for delirium (Finucane et al. 2020; Bramati and Bruera 2021) and ELDVs (Grant et al. 2023), ensures patient safety and comfort while honoring their experiences.

Clinical illustrations:

- **Example A (delirium alone):** A patient reports hostile “people in the room,” is disoriented, highly agitated, and cannot coherently narrate the experience. The content is nonbiographical and persistently threatening. Prioritize delirium management and safety.
- **Example B (ELDV alone):** A lucid patient near death describes peaceful bedside visits from loved ones, remains oriented, and shows reduced anxiety after these experiences. No CAM features are present. Provide supportive presence and follow guidelines described in Grant et al. (2023).
- **Example C (dual presentation):** A patient is CAM positive with fluctuating attention and altered state of consciousness. In some windows of lucidity, she consistently recounts vivid encounters with a deceased spouse, thematically focused on leave-taking. She expresses distress with the experience because she does not feel prepared to die. Treat the delirium precipitants; avoid automatically suppressing the consolidating ELDV. Use supportive interviewing to honor its meaning while monitoring risk.

## Conclusions and future directions

This complex case underscores the intricate nature of mental states changes experienced by patients in the final stages of life and the considerable challenges inherent in their clinical management. The patient exhibited fluctuating states of delirium and distressing ELDVs. It remains an open question whether the patient’s inability to derive the comfort typically associated with such experiences stemmed from the effects of delirium, a preexisting depressive state rooted in a life marked by resentments, or the pervasive psychological, existential, and spiritual suffering often present in the dying process – or indeed, from a multifactorial interplay of these elements.

The thematic content and qualitative characteristics of ELDVs are crucial for distinguishing them from delirium. For instance, ELDVs often involve specific themes (e.g., deceased loved ones, travel, transitions), retain a subjective sense of clarity, and can convey meaning, even when distressing. In contrast, hallucinations associated with delirium are typically more fragmented, less coherent, and often linked with nonspecific fear or disorientation, as evidenced by the Japanese study which reported diverse and often catastrophic, hallucination content (Tachibana et al. 2021). While certain distressing emotions are clearly linked to delirium, their precise connection to ELDVs or the physical decline of the dying process remains uncertain, suggesting that emotional valence may not serve as a definitive discriminator between ELDVs and delirium (Machado and Moreira-Almeida 2021; Silva et al. 2023), especially given the significant influence of cultural and psychosocial factors on their emotional impact (Maraldi et al. 2024).

Our findings also suggest that CAM may lack sufficient sensitivity to distinguish effectively between ELDVs and delirium when both are concurrently present, particularly in instances involving fluctuations in attention or pronounced emotional distress. From a clinical perspective, the accurate differentiation of these phenomena is paramount. Misdiagnosing ELDVs as delirium carries the risk of suppressing important psychological and spiritual processes vital to the patient’s end-of-life journey. Conversely, overlooking delirium can prolong suffering and intensify caregiver burden (Barnes et al. 2010; Featherstone et al. 2021).

Effective clinical management in such complex cases requires the integration of subjective aspects of the dying process with conventional biomedical treatment, thereby ensuring holistic care that comprehensively addresses the broader needs of the dying individual (Puchalski 2002; Kerr and Mardorossian 2020). High-quality palliative care, which encompasses effective management of distressing symptoms at end of life, especially delirium, alongside person-centered care that includes attention to the patient’s psychosocial and spiritual experiences, is essential for ensuring a dignified and meaningful death (Featherstone et al. 2021; Pan et al. 2021). This is particularly noteworthy given that delirium is often underrecognized by palliative care teams (Barnes et al. 2010), despite its significant distressing impact on both patients and their families (Breitbart and Alici 2008; Cohen 2015).

Current research demonstrates a limited body of evidence regarding nonpharmacological management of delirium in palliative care settings, and a scarcity of clinical trials examining pharmacological interventions (Featherstone et al. 2021). Similarly, distressing ELDVs also remain poorly understood, highlighting the urgent need for further research into their prevalence, types, meanings, and effects (Claxton-Oldfield 2023). Consequently, there is a distinct absence of established guidelines or best practices for the management of the distressing ELDVs (Claxton-Oldfield 2023; Silva et al. 2023).

Therefore, this case highlights a critical need for: (1) systematic longitudinal assessments of mental states at the end of life; (2) the development and validation of tools specifically designed to capture ELDVs; and (3) comprehensive interdisciplinary training for healthcare providers. Further research is imperative to refine classification criteria, explore the cultural variability of ELDVs, and identify best practices for managing distressing cases.

In conclusion, ELDVs and delirium, while phenomenologically distinct, may co-occur and overlap in late-stage illness. Their differentiation is nuanced and context-dependent. A deeper understanding of their coexistence has the potential to significantly improve diagnostic accuracy, enhance patient comfort, and elevate the overall quality of the dying experience.

To address this significant clinical and educational gap, methodologically rigorous, bold, and innovative studies are essential. This includes, but is not limited to, mixed-method and longitudinal studies comparing dreams and visions in patients with and without delirium, which would be valuable in advancing our understanding of human consciousness during the dying process. Furthermore, future research should aim to develop a standardized scale for identifying subjective reports of ELDVs. Concurrently, investing in research focused on effective strategies for improving healthcare professionals’ clinical skills training is crucial to ensure higher quality care at the end of life.

## Supporting information

10.1017/S1478951525101247.sm001 Silva et al. supplementary material 1 Silva et al. supplementary material

10.1017/S1478951525101247.sm002 Silva et al. supplementary material 2 Silva et al. supplementary material

10.1017/S1478951525101247.sm003 Silva et al. supplementary material 3 Silva et al. supplementary material

## Supplementary material

The supplementary material for this article can be found at https://doi.org/10.1017/S1478951525101247.
