# Supplementary material for: End-of-life dreams and visions in a patient with delirium: A Brazilian case report and narrative review
Source: Palliat Support Care. 2025 Dec 26;24:e4. doi: 10.1017/S1478951525101247 (PMC13166248; doi:10.1017/S1478951525101247)
Supplement: Silva et al. supplementary material 1 — Silva et al. supplementary material [file S1478951525101247sup001.docx]

**SUPPLEMENTARY MATERIAL I**

**MONITORING OF MRS. DAYANE’S MENTAL STATE AT THE TIME OF REPORTING THE EXPERIENCES**

|  | | | | | |
| --- | --- | --- | --- | --- | --- |
| **CAM** | **State of Consciousness** | **Experiences Transcriptions** | **Interpretation**  **(“What is the meaning of this dream/experience for you?” **)** | **Emotional Quality** | **Day of Admission** |
| SR | Asleep | **Mrs. Maria (Daughter):** *Hmm... She... Fell asleep on the couch here, and she woke up, yeah, saying: “ah, I dreamed about mom...” Yeah... She said it was good, like that, and... I-I don't remember the details, but I think that yes, they were in a good place, like that... From what I understood.* | **Mrs. Maria (Daughter):** *So, I think that... It's the unconscious, trying to... Conform to the situation. ... This is something very laymanlike.* | Comforting | 3 months before admission |
| **-** | Asleep and as soon as she woke up | **Mrs. Dayane (Patient):** *I dreamed about... That my niece had a baby. A little girl. The cutest, most beautiful, most beautiful thing. I fell in love. Then she... She stood up for me, you know? And then, I was so happy, I picked her up in my arms and lifted her up. Then, when I stood up, she did this, I woke up with her in my arms.* | **Mrs. Dayane (Patient):** *I think it's a sign of more life for me... And hope.* | Comforting | 25^th^ |
| **+** | Awake | **Mrs. Maria (Daughter):** *She started calling for her ex-sister-in-law, who is also deceased.*  **Mrs. Veronica (Sister):** *But she didn't have much contact.*  **Mrs. Maria (Daughter):** *And she started calling for my grandmother, and lately she has been talking a lot about her grandmother, who had the same disease as her. And... And they are very similar in that sense. But she called a lot, she started talking about things in the past. Yes, I think about situations that may have happened to her grandmother, and she also said: 'Mom, let me open the door, I need to go', like that.* | Mrs. Maria (Daughter) reported that after the oncologist confirmed that her disease was irreversible, Mrs. Dayane began to get worse, presenting this episode five days after the consultation. For her, the patient calls for her mother because she has the same disease that she had. | Distressing | 28^th^ |
| **+** | Asleep  (#1)  Awake  (#2, #3 and #4) | **#1**  **Psychologist:** [...] *we arrived for the... appointment, she was more, uh ..., sleepy, she wasn't, uh, having much social interaction, right... It even seemed like she was in a more comatose state, but from the moment, right, when she was approached, when she was asked how she was... She said that she had died that night, right, and that she had come back from that death, right. That she had, let's say, been resurrected, right? (...) Uh, and that she was very afraid of dying, of death, so much so that she called all her family ... All her brothers, she was afraid... That her sister who lives a little further away wouldn't arrive, because she said several times that her sister was taking a long time, right. She called ... She asked a pastor to come in the afternoon, right? to be able to talk to her... So, in a way it seems like she is preparing herself, right, and making all the necessary arrangements, right? with her family, her daughter who is a little less present was present at that moment, right. Her sisters were there, there were... Two or three (?), now I don't remember if there were two or three sisters. And the other one who lives... I believe she lives a little further away, I also don't know where she lives, right, uh ..., she was coming, so calling also, a matter of being able to talk to this religious leader, right, so it seems like she is making all the necessary arrangements for, for her departure.*  **#2**  **Psychologist:** And how did this strange thing happen? (...)  **Patient:** A very bad thing... (...) I screamed a lot; I screamed a lot. (...)  **Psychologist:** So, it was a really bad feeling?  **Patient:** Very bad... (...)  **Psychologist:** Really? What did you feel?  **Patient:** Very scared. (...)  **Psychologist:** But are you still scared? No? Is it over?  **Patient:** Hmm... It's been a little while. (...)  **TOS (Researcher):** Did you see something or dream about something that made you scared?  **Patient:** No, no, no. (...)  **Psychologist:** And what can we do to help you?  **Patient:** Nothing.  **Psychologist:** Nothing?  **TOS (Researcher):** Not even to help reduce this fear?  **Patient:** No. (...) I impose a lot of fear on myself.  **Psychologist:** Did you impose a lot of fear on yourself?  **Patient:** Uh-huh.  **Psychologist:** And why did you do that?  **Patient:** I don't know, I don't want to know. (...)  **Psychologist:** Uh-huh ... And we understand. And we respect you.  **#3**  **Mrs. Maria (Daughter):** *She kept talking to her grandmother, and today she spoke about a sister of hers who had passed away and had come to see her, and that she wanted her to come here, to her house. And that she wanted to know how she was going to get up... She used the term “get up”. (…) and she also asked for (...) a pastor to come, because... she seems to want to say goodbye and is worried about the afterlife. (...) She also asked about my father when she died. But it seems that she talked to him like she says she talked to her grandmother.*  **#4**  **Mrs. Veronica (Sister)**  *She said she was afraid of dying, that she was... That she didn't want to die, that she didn't want to die, that she was afraid. That she wasn't anymore, because "I don't know, I don't know if you're going with me." I said "I'm going, I'll go to the door and hand it over to you," "No, no, I want to go with you, I want you to go with me," "How are we going?" She said "Well, it's up to God to know how you're going... But I'll only go if you go, don't leave my side" (...) Then I said "Ok, then I'll go, I'll go there and come back." "But are you really going?" Then I said "yes." Then today she asked me about a sister who had already died. Then I said "Oh, she turned into a little star...". (...) "No, no, no, no, she didn't turn into anything." I said "Well, if you go, she... you 'll meet her." “No, but I don’t want to yet” (...) “But why are you afraid to go, isn’t it a good place?”, “I didn’t want to go yet” “But you’re tired, aren’t you?”, “Yeah, I am” “So...” I say: “But it’s in your time, not ours”. (...) “Whenever you think you have time, I’ll be with you and you’ll go”, I say, “I can even go with you wherever you want”. “Oh, okay then, if you’re going, I’ll go”, and now she asks all the time “Shall we go?”.*  **#5**  **Mrs. Verônica (Sister):** *Yesterday she said: “I died, right? Say it’s a lie that I died.” (...) “I did die, you’re crying.” I say: No, look at me, I ’m here, look, touch me, I ’m warm, I’m not cold. “No, I did die, you don’t want to tell me that I died? I ’m in the hospital.” I say: You’re not in the hospital, you’re in Maria’s house, you’re alive. It’s not your time yet, you’re the one who’s going to decide when your time is. (...) And the other one [sister] is coming now, come now. (...) She’s anxious for her to arrive, to tell the truth. “Tell me the truth,” “what’s the truth?”, “that I did die, you’re crying.” (...) Then she sees that she didn’t and laughs. “Okay,” she says, “okay.”* | **Psychologist:** *So, when we arrived, it seemed like she had a very low level of consciousness?... But when she spoke, it seemed like she was very lucid, very lucid and it was specifically in relation to this lucidity, to this specific point, this context of life and death.*  **Mrs. Veronica (Sister):** *I think it's a little bit due to the medication and the process of evolution, like that. She's taking the morphine, right, and suddenly she's hallucinating. (...) But the rest I think is that, because the mother was like that, between there and here, but the mother was in pain, she doesn't have pain now.* | Distressing | 29^th^ |
| **-** | Awake | **#1**  **Mrs. Maria (Daughter):** *She hallucinated, she was quite delirious, she was quite agitated, yeah... And that night in particular it was more, more, oh, I think she slept for five minutes at most and woke up talking; that was it during the night. She talked a lot, but we couldn't understand, what we could understand was the names of her brothers, who she is very close to, and she, we couldn't understand what she was saying, but she would raise her hands like this and say "I don't know what, Duda..." and the rest she spoke in a jumble. It seems like she's in her childhood, yeah, she talks about a lot of things from her childhood, so...*  **#2**  **Mrs. Maria (Daughter):** *Yes, she said there was a little girl with curly hair on the swing, but I wasn't there, it was my sister* [**Laura**]*. Then my sister asked if it was me, because I have curly hair, she said “no, it's another little girl”. Yes, and she was talking laughing to this little girl. And then when she looked at the middle of the wall, she got very scared, she said: “oh, the animals are coming to get me”, she said.*  **#3**  **Nurse:** *Who is Tereza she's talking about?*  **Mrs. Maria (Daughter):** *She's been talking about Teresa since yesterday, she's been saying some names that I don't know either. There are some names that...*  **Mrs. Laura (Daughter):** *She asked if I had seen Teresa. ... Then I said “no, I don’t know that one”.*  **Mrs. Maria (Daughter):** *Neither.* | **Mrs. Maria (Daughter):** *Yes, it's just that now, after she became aggressive, she came back speaking more clearly... recognizing us, but she wasn't recognizing us, she was calling me Julia, which is the name of one of her sisters. And now she's calmer, at least calm and what she says is understandable.* | Comforting and distressing | 32^nd^ |
| SR* | Awake | **Mrs. Veronica (Sister):** *She often sees a child with curly hair, either riding on a swing or laughing at her, and yesterday she called me and said she was petting the child. So, who is she? “A child, such a cute little baby” and she laughed and ran her hand over her and said: “Take it, take it, take it for me because my hands are so heavy and I’m going to drop it.” Then she reached for me, the child like that, and she said “see how cute” and laughed, like that, then she changed and didn’t want it anymore either... It was quick and it passed.* |  | Comforting | 33^rd^ |
| **+** | Asleep | **Mrs. Verônica (Sister):** *It was quiet at the beginning of the night, but after a while, she started screaming for my sister who had already passed away. She screamed a lot and said “come, come”, and called her, and then she stayed like that for a long time, hallucinating and calling her and raising her hand and calling her. Then she said “no, no, no, no”, I don’t know if she didn’t want to leave where she was ... It seemed like she didn’t want to go. Because when she was sleeping, she put her hand up like this, smiles, and then waves it. Then we ask “what happened?” “There, there she is”. Then she keeps smiling like that, and soon she passes out.* |  | Neither comforting nor distressing | 36^th^ |

**Subtitles:** SR: no record / * Patient was sleeping / ** This question was asked during the interviews, after the dream/experience was reported.
