# Supplementary material for: End-of-life dreams and visions in a patient with delirium: A Brazilian case report and narrative review
Source: Palliat Support Care. 2025 Dec 26;24:e4. doi: 10.1017/S1478951525101247 (PMC13166248; doi:10.1017/S1478951525101247)
Supplement: Silva et al. supplementary material 2 — Silva et al. supplementary material [file S1478951525101247sup002.docx]

**SUPPLEMENTARY MATERIAL II**

**Results of Additional Tests**

**(Day 29 of the follow-up)**

| Lactate Dehydrogenase (RI: 125 to 220 U/L) | 267 U/L |
| --- | --- |
| Alanine Aminotransferase – ALT (RI: up to 33 U/L) | 22 U/L |
| Aspartate Aminotransferase – ALT (RI: 5-50 U/L) | 24 U/L |
| Alkaline Phosphatase (RI: 83-248 U/L) | 210 U/L |
| Gamma-Glutamyl transferase – GGT (RI: 38 U/L) | 170 U/L |
| Potassium (RI: 3.5 to 5.1 mmol/L) | 4.3 mmol/L |
| Sodium (RI: 136 – 145 mmol/L) | 137 mmol/L |
| Creatinine (RI: 0.5- 0.9 mg/dL) | 0.45 mg/ dL |
| Erythrocytes (RI: 3.8 – 4.8 million µ/dL) | 3.49 million µ/L |
| Hemoglobin (RI: 12 – 15 g/dL) | 11.1 g/ dL |
| Total Leukocytes (RI: 4000 – 10000/mm2) | 5440 |
| Platelets (RI: 323000/mm2) | 323000 mm2 |
