# Supplementary material for: End-of-life dreams and visions in a patient with delirium: A Brazilian case report and narrative review
Source: Palliat Support Care. 2025 Dec 26;24:e4. doi: 10.1017/S1478951525101247 (PMC13166248; doi:10.1017/S1478951525101247)
Supplement: Silva et al. supplementary material 3 — Silva et al. supplementary material [file S1478951525101247sup003.docx]

**SUPPLEMENTARY MATERIAL III**

**Medications with Dosage and Prescription Date***

| MEDICATIONS | 9^th^ day | 21^st^ day | 23^rd^ day | 29^th^ day | 37^th^ day |
| --- | --- | --- | --- | --- | --- |
| Methadone 10mg (PO) | (1/2-1-1) CMP | (1-1-1-1) CMP  + 1/2 SOS - up to twice | (1-1/2-1) CMP | (1-1-1-1) CMP  + 1/2 SOS - up to twice |  |
| Dipyrone 500mg (PO) | (2-2-2-2) CMP | - | (2-2-2-2) CMP | (2-2-2-2) CMP |  |
| Dipyrone 2mL - 500mg/ml (SC) | - | (1-1-1-1) AMP | - | (0-1-0) CMP |  |
| Dexamethasone 4mg (PO) | (1-0-1) CMP | (1-0-1) CMP | (1-0-0) CMP | - |  |
| Ondansetron 8mg (CMP) | (1-1-1) CMP | - | (1-1-1) CMP | (1-1-1) CMP |  |
| Dimenhydrinate 50mg + Pyridoxine 10mg (PO) | (1-1-1) CMP | (1-1-1) CMP | (1-1-1) CMP | (1-1-1) CMP |  |
| Sertraline 50mg (PO) | (0-2-0) CMP | (0-2-0) CMP | (0-2-0) CMP | (0-2-0) CMP |  |
| Phenytoin 100mg (PO) | (1-1-1) CMP | (1-1-1) CMP | (1-1-1) CMP | (0-1-0) CMP |  |
| Folic Acid 5mg (PO) | (0-1-0) CMP | (0-1-0) CMP | (0-1-0) CMP | (0-1-0) CMP |  |
| Lactulose 120mL - 667mg/mL (VO) | (20mL- 20mL- 20mL) | - | - | - |  |
| Metoclopramide 2mL - 5mg/ml (SC) | - | (1-1-1) AMP | - | - |  |
| Metoclopramide 10mg (PO) |  |  | (1-1-1) CMP | - |  |
| Glycerin Solution 120mg/mL (VR) | - | 1 FR 1/1 weeks | 1 FR 1/1 weeks | 1 FR of 1/1 weeks |  |
| Risperidone 1mg (PO) | - | (0-0-1) CMP | (0-0-1) CMP | (0-0-1) CMP |  |
| Clonazepam 0.5mg (PO) | (0-0-1) CMP |  | - | - |  |
| Clonazepam 20mL - 2.5mg/ml (PO) | - | (0-0-10) DROPS | (0-0-10) DROPS | (0-0-10) DROPS |  |
| Bisacodyl 5mg (PO) | - | (0-1-0) CMP | (0-1-0) CMP | (0-1-0) CMP |  |
| Alprazolam 0.5mg (PO) | - | - | (0-0-1) CMP | (0-0-1) CMP |  |
| Gabapentin 300mg (PO) | - | - | - | (0-1-0) CAP |  |
| Haloperidol 1mL - 5mg/mL (SC) | - | - | - | (1-1-1) AMP | (1-1-1) AMP |
| Chlorpromazine 25mg (SC) | - | - | - | (1-1-1) AMP | (1-1-1) AMP |

* Medication prescriptions were weekly and only changed with changes in the clinical condition.

Abbreviations: AMP: ampoule; CMP: tablet(s); FR: vial; SC: subcutaneous; SOS: rescue medication, use only in the event of acute symptoms; VO: oral route; VR: rectal route.

The numbers in parentheses, (1-0-1) CMP for example, refer to the intake of medication in relation to the periods of the day. In the example given, it would be 1 tablet every 12 hours.
